# Trace element profiles of the sea anemone *Anemonia viridis* living nearby a natural CO_2_ vent

**DOI:** 10.7717/peerj.538

**Published:** 2014-09-09

**Authors:** Rael Horwitz, Esther M. Borell, Maoz Fine, Yeala Shaked

**Affiliations:** 1 The Mina and Everard Goodman Faculty of Life Sciences, Bar-Ilan University, Ramat-Gan, Israel; 2 Institute of Earth Sciences, The Hebrew University, Jerusalem, Israel; 3 The Interuniversity Institute for Marine Sciences, Eilat, Israel

**Keywords:** Ocean acidification, Trace elements, Metals, CO_2_ vent, *Anemonia viridis*

## Abstract

Ocean acidification (OA) is not an isolated threat, but acts in concert with other impacts on ecosystems and species. Coastal marine invertebrates will have to face the synergistic interactions of OA with other global and local stressors. One local factor, common in coastal environments, is trace element contamination. CO_2_ vent sites are extensively studied in the context of OA and are often considered analogous to the oceans in the next few decades. The CO_2_ vent found at Levante Bay (Vulcano, NE Sicily, Italy) also releases high concentrations of trace elements to its surrounding seawater, and is therefore a unique site to examine the effects of long-term exposure of nearby organisms to high *p*CO_2_ and trace element enrichment *in situ*. The sea anemone *Anemonia viridis* is prevalent next to the Vulcano vent and does not show signs of trace element poisoning/stress. The aim of our study was to compare *A. viridis* trace element profiles and compartmentalization between high *p*CO_2_ and control environments. Rather than examining whole anemone tissue, we analyzed two different body compartments—the pedal disc and the tentacles, and also examined the distribution of trace elements in the tentacles between the animal and the symbiotic algae. We found dramatic changes in trace element tissue concentrations between the high *p*CO_2_/high trace element and control sites, with strong accumulation of iron, lead, copper and cobalt, but decreased concentrations of cadmium, zinc and arsenic proximate to the vent. The pedal disc contained substantially more trace elements than the anemone’s tentacles, suggesting the pedal disc may serve as a detoxification/storage site for excess trace elements. Within the tentacles, the various trace elements displayed different partitioning patterns between animal tissue and algal symbionts. At both sites iron was found primarily in the algae, whereas cadmium, zinc and arsenic were primarily found in the animal tissue. Our data suggests that *A. viridis* regulates its internal trace element concentrations by compartmentalization and excretion and that these features contribute to its resilience and potential success at the trace element-rich high *p*CO_2_ vent.

## Introduction

Increasing carbon dioxide (CO_2_) emissions drive ongoing ocean acidification (OA) and place marine ecosystems at a vulnerable state (Hoegh-Guldberg, 2012). The growing consensus is that there is a need to understand the impact of multiple stressors in combination with OA on marine organisms rather than examining acidification alone (Denman et al., 2011). The synergistic effects of declining pH/rising *p*CO_2_ with environmental pollutants have received little attention to date. Trace metal contamination occurs in many near-shore coastal environments due mainly to anthropogenic inputs such as industrial effluent and run-off (Brock & Bielmyer, 2013). In excess, these trace metals may cause adverse biological effects in marine organisms (Dallinger & Rainbow, 1993), and moreover, in combination with OA these effects may be exacerbated (Lewis, Clemow & Holt, 2013).

Natural CO_2_ vents in sub-tropical coastal areas are recognized as natural laboratories to study long-term effects of elevated *p*CO_2_ (pH) across many biological and spatial scales (Hall-Spencer et al., 2008; Kroeker et al., 2011; Meron et al., 2012). Such a location exists in the Levante Bay of Vulcano Island (Italy) in the Mediterranean Sea, where several studies have examined physiological responses of marine organisms to OA conditions (Johnson et al., 2011; Arnold et al., 2012; Johnson et al., 2012; Lidbury et al., 2012; Suggett et al., 2012; Calosi et al., 2013; Borell et al., 2014). The submarine gas emissions from the cold, shallow seeps in Levante Bay are characterized by high CO_2_ contents volume (>90%) and variable H_2_S (ranging 0.8 to 2.5% volume) (Capaccioni, Tassi & Vaselli, 2001; Boatta et al., 2013). Recent reports found significant enrichment of trace metals in seawater and sediments as compared to areas far from the vent site (Kadar et al., 2012; Boatta et al., 2013; Vizzini et al., 2013). This unique site, therefore, offers conditions of long term exposure of the biota to a myriad of trace metals (which we will refer to as trace elements hereon) and OA-like conditions, a situation that is hard to replicate in a laboratory study.

Trace elements are bioconcentrated in marine invertebrates by at least one order of magnitude when compared to seawater (Eilser, 1981; Simkiss & Taylor, 1989). Some trace elements are beneficial for the organism metabolism, while others are toxic (Rainbow, 2002). Essential trace elements, such as iron (Fe), manganese (Mn), copper (Cu) and zinc (Zn), are vital components of enzymes and play an important role in electron transport reactions (White & Rainbow, 1985). Other trace elements, for example—cadmium (Cd), lead (Pb) and arsenic (As), lack any known biological role in marine invertebrates and exhibit high toxicity if allowed to accumulate at metabolically-active sites (Depledge & Rainbow, 1990; Kohlmeyer, Kuballa & Jantzen, 2002; Rainbow, 2002).

Extensive research has focused on the potential impact of OA on marine organisms, particularly tropical reef-building corals. However, non-calcifying Anthozoans, such as sea anemones, have received less attention (Towanda & Thuesen, 2012). *Anemonia viridis* is a temperate Mediterranean species, which occurs naturally throughout Levante Bay. The anemones show no apparent signs of stress to the prevalent environmental conditions close to the primary vent (Suggett et al., 2012). Recent studies have reported no change in anemone tissue protein, algal symbiont densities and their chlorophyll concentrations (Borell et al., 2014), but also increased growth (abundance and size) and enhanced photosynthetic rates of *A. viridis* at the site proximate to the vent (Suggett et al., 2012).

Trace element regulation in marine invertebrates involves excretion, impaired uptake, detoxification and storage (Depledge & Rainbow, 1990). In different anemone species, higher body loads were recorded upon exposure to elevated but non-lethal levels of dissolved trace elements, such as Cu, Cd and Zn (Harland & Nganro, 1990; Harland, Bryan & Brown, 1990; Mitchelmore et al., 2003; Mitchelmore, Ringwood & Weis, 2003; Main, Ross & Bielmyer, 2010; Brock & Bielmyer, 2013). Upon transfer to clean seawater the levels of trace elements in the anemones dropped, indicative of active excretion (Mitchelmore et al., 2003; Brock & Bielmyer, 2013). In some cases anemones, subjected to high trace element concentration, were reported to release their symbiotic algae (termed “bleaching”; Evans, 1977; Harland & Nganro, 1990; Mitchelmore et al., 2003; Main, Ross & Bielmyer, 2010; Brock & Bielmyer, 2013). As a result the concentrations of trace elements associated with the algae were shown to drop (Harland & Nganro, 1990), most likely via the tentacles where the majority of algae reside (Glider, Phipps & Pardy, 1980). Several researchers have suggested algae expulsion is used as a mechanism of metal detoxification, since algae have been found to accumulate metals to a larger extent and be more tolerant than their symbiotic hosts (Peters et al., 1997; Jones, 2004). Other suggested mechanisms for regulating trace elements in anemones include sequestration of metals into granules (Van-Praet, 1977; Gupta & Hall, 1984) and release via increased production of mucus or nematocyst discharge (Gupta & Hall, 1984; Tardent et al., 1990). Metal-binding to proteins such as glutathione (GSH) (Mitchelmore, Ringwood & Weis, 2003) and metallothioneins (MT) (Mitchelmore et al., 2003) may also play a role in reducing the tissue metal burdens in these animals.

The increase in trace element concentrations nearby the vent most likely results from metal enriched fluid and/or gas emissions from the seeps themselves, higher metal solubility due to elevated *p*CO_2_, and increased fluxes of sediment-bound metals (Millero et al., 2009; Kadar et al., 2012; Roberts et al., 2013; Vizzini et al., 2013). In addition to the total trace element concentration in an ecosystem, their speciation (namely the presence of free ions) determines their biological availability or potential toxicity (Newman & Jagoe, 1994). Elevated *p*CO_2_ levels and/or low pH in seawater can increase the bioavailability of trace elements due to increased solubility and/or changes in the metal speciation (Byrne, Kump & Cantrell, 1988; Ardelan et al., 2009; Millero et al., 2009; Lopez et al., 2010; Millero & DiTrolio, 2010). Increased *p*CO_2_ concentrations can also affect the uptake/excretion mechanisms of trace elements in marine organisms (Lacoue-Labarthe et al., 2009; Lacoue-Labarthe et al., 2011; Pascal et al., 2010; Fitzer et al., 2013; Ivanina et al., 2013; Lewis, Clemow & Holt, 2013; Götze et al., 2014). It is, therefore, of interest to investigate the anemone trace element content along the natural CO_2_ gradient and probe for accumulation, compartmentalization, or excretion.

The simultaneous exposure of *A. viridis* to multiple trace elements in high CO_2_ surroundings presents us with a unique opportunity to bring new insights into trace element regulation under OA-like conditions *in situ*. The aim of our study was to compare *A. viridis* trace element profiles and compartmentalization between high *p*CO_2_ and control environments. We hypothesized that *A. viridis* living in low-pH waters near the primary vent in Levante Bay bioaccumulate trace elements to a different extent than those in the neighboring ambient pH environment, and that the bioaccumulation patterns are different between body compartments. To test our hypothesis we collected and analyzed anemones growing next to the vent and at a control site. Rather than examining the whole anemone body, we analyzed the trace element partitioning between tentacles and pedal disc and within the tentacles between the algal symbiont (i.e., *Symbiodinium*) and animal tissues.

## Materials and Methods

### Study sites

**Figure 1:**
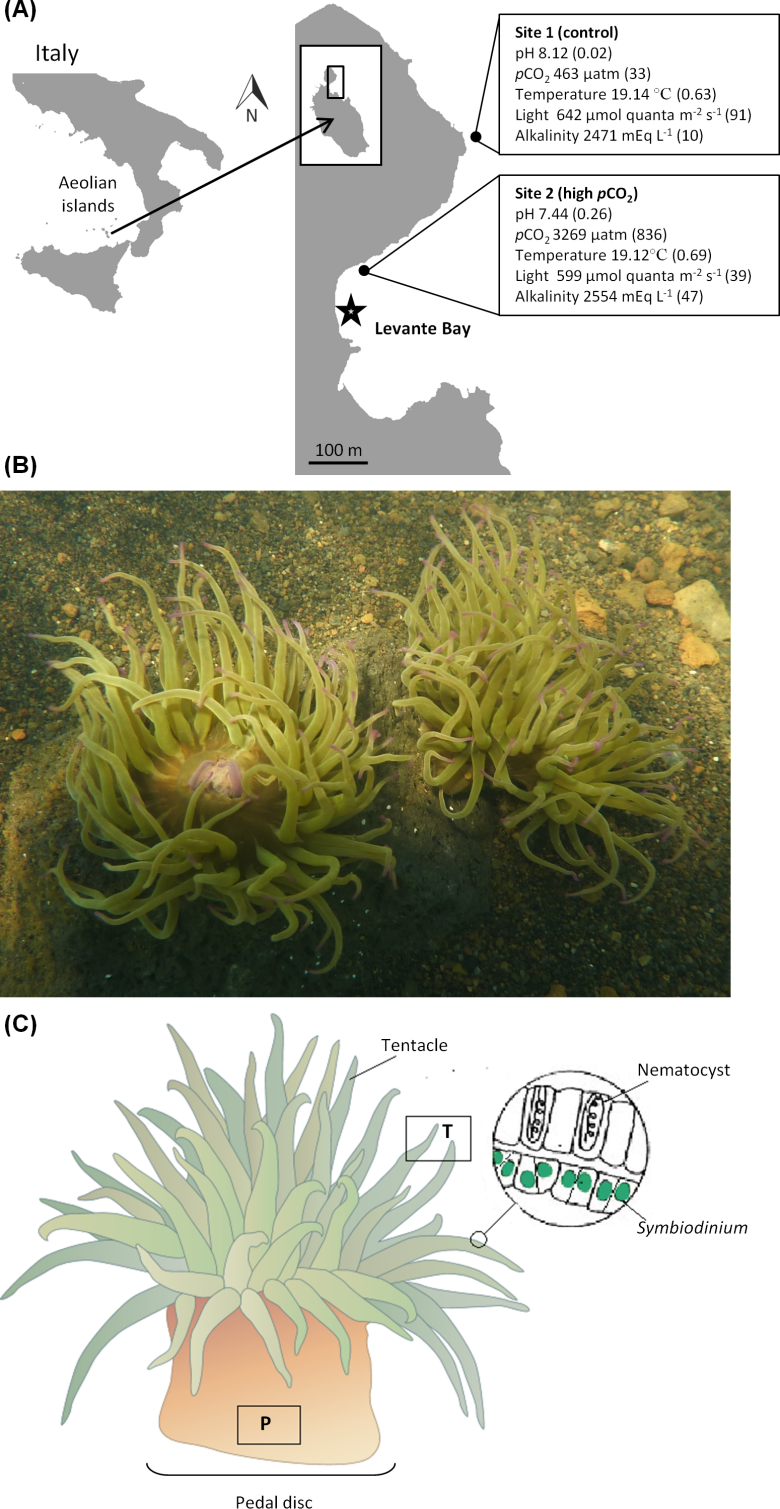
General information on the study sites, studied organism and sampling procedure. (A) Map of the study area with sampling sites 1 (control) and 2 (high *p*CO_2_). Boxes show mean values (± SD) of each site for: pH, *p*CO_2_, temperature, light and alkalinity. The primary vent is indicated with a star symbol. (B) Image showing *A. viridis* at site 2 (high *p*CO_2_). (C) Diagram of *A. viridis* showing the body compartments excised for trace element analysis: (T) tentacles and (P) pedal disc. Tentacles were separated to symbiont algae (*Symbiodinium*) and animal tissue for further analysis.

This study was conducted along the sub-littoral in Levante Bay, Vulcano Island (38°25′N, 14°57′E), part of the Aeolian Island chain, NE Sicily (Fig. 1A) in May 2012. Shallow-water CO_2_ vents create a natural CO_2_/pH gradient along the north-easterly side of the bay ranging from pH 6.05 to 8.29 at >350 m from the vent site (Johnson et al., 2011; Boatta et al., 2013). The vents also create a gradient of trace elements in the seawater and the sediments, where elevated trace elements are recorded up to 300 m away from the vent (Boatta et al., 2013; Kadar et al., 2012; Vizzini et al., 2013). Two sites were selected for sampling (Fig. 1A); Site 1 (control) was an ambient seawater reference station, located outside the vent area influence (>400 m), whereas site 2 (high *p*CO_2_) was in proximity to the CO_2_ vents (∼260 m) in accordance with previous studies (Johnson et al., 2011; Arnold et al., 2012; Suggett et al., 2012; Borell et al., 2014).

### Sample collection

#### Anemones

*A. viridis*, a dominant benthic organism in Levante Bay, was prevalent throughout the study area (Fig. 1B). Twenty anemones were haphazardly collected from each site at a depth of 1–2 m and immediately frozen until further analyses. To minimize any confounding responses due to age and/or size (Perez, Cook & Brooks, 2001) all samples were of similar size (oral disc diameter of 2.5–3.5 cm).

#### Suspended particulate material

Anemones may obtain trace elements from their prey or acquire dissolved metals from the surrounding seawater (Harland & Nganro, 1990; Harland, Bryan & Brown, 1990). The trace element content of the anemone’s potential prey was assessed by collecting plankton and other suspended particulate material from seawater. Plankton hand nets (50 cm mouth diameter, 100 µm mesh size) were towed at each site for 15 min. The collected material was gently transferred to sterile trace metal clean 50 ml test tubes (Stardest) and cooled until further analysis.

### Physicochemical properties of the seawater

At both sites seawater pH (NBS scale) and temperature were measured several times a day for 4 days using a pH meter (YSI Professional Plus, Handheld Multiparameter Instrument). Water samples for total alkalinity (TA) analyses were collected from each site, cooled and stored in the dark until analysis. TA was quantified with a Metrohm 862 compact titrosampler (Cohen, 2011). The *p*CO_2_ levels were calculated from pH_NBS_ measurements, TA and salinity (Johnson, 2012), using the program CO2 SYS (Pierrot, Lewis & Wallace, 2006), selecting the constants of Mehrbach et al. (1973). Light intensity was measured hourly for 3 consecutive days close to the seabed (1–2 m depth) with HOBO Pendant^®^ Temperature/Light data loggers (Onset, Pocasset) and then converted from lux to µmol quanta m^−2^ s^−1^ (Thimijan & Heins, 1983).

### Sample preparation

#### Trace element clean procedures

Special care was taken to minimize sample contamination during handling. Samples were treated in a trace element clean facility (class 10,000) equipped with three class 100 laminar flow hoods, using double distilled water (DDW, Milli-Q 18.2 *M*Ω; MQ Academic) and double distilled acids. All test tubes were trace-element clean certified (Stardest) and other lab ware was thoroughly cleaned with acids (10% HCl (analytical grade, Merck) for 24 h following a thorough rinse in DDW water). Solutions were prepared with DDW water and analytical grade or higher purity salts.

#### Dissection of tentacles and pedal disc tissues

To examine the distribution of trace elements in the anemone body, two distinct body compartments were excised and analyzed separately (Fig. 1C). Duplicate subsamples of ∼0.25 g were cut with a new razor from both the tentacles (*n* = 10 per site) and the pedal disc (*n* = 10 per site) of each anemone. The subsamples were placed in pre-weighed trace element clean 15 ml test-tubes and their wet weight recorded with a sensitive scale (CT 1202, Citizen, accuracy 0.0001 g).

#### Separation between Symbiodinium and animal tissue

To further examine the partitioning of trace elements between the symbiotic algae and anemone host tissue, duplicate subsamples of ∼1 gr were excised from the tentacles of each anemone (*n* = 5 per site). The subsamples were placed immediately in a trace element clean pre-weighed 15 ml test tube and their wet weight recorded. One duplicate was digested in acid to yield the total tentacle trace element concentration. The other duplicate received 5 ml NaCl solution (35 g L^−l^) and was homogenized for 3 min with an electric homogenizer (DIAX 100 homogenizer; Heidolph Instruments). The homogenate was centrifuged for 5 min at 5,000 rpm (4K15 centrifuge; Sigma) to separate the *Symbiodinium* (pellet) and the host tissue (supernatant). Visual inspections revealed no crossover of material between these components, but both were washed carefully. The host supernatant was homogenized and centrifuged for 10 min at 13,500 rpm. Then, the pelleted host material was washed three times by means of re-suspension in 3 ml NaCl solution (20 g L^−l^) and centrifuged for 5 min at 13,500 rpm. The *Symbiodinium* pellet was washed three times by means of re-suspension in 2 ml NaCl solution (20 g L^−l^) and centrifuged for 3 min at 5,000 rpm.

### Digestion and analysis

Samples were digested within the test tubes in the presence of 5–10 mL double distilled 9.8 N HNO_3_. The closed lid test tubes were placed in acid cleaned glass beakers filled with DDW that were submerged in a water bath and heated to 80–90 °C for 1–7 days. Special care was taken to avoid contact between the outside water and the upper part of the test tube. Once the digested material lost its texture and color and appeared clear, the samples were cooled and supplemented with DDW. Samples with expected high trace element content were also diluted by 5–50 fold by transferring aliquots into new test tubes. Analysis of trace element content (and macro elements) was carried out using an Agilent 7500cx ICP-MS (Agilent Inc.). A set of calibration standards was added to the analysis together with appropriate blanks. An internal standard solution containing Sc, Re and Rh was introduced during the analyses to all standards and samples. The reported values are blank subtracted and averaged over duplicate analysis, with difference in % between duplicate analyses typically smaller than 10%. Results are given in ng trace element per mg dry weight (wt) of anemone.

### Statistical analyses

Differences between pedal disc and tentacles in each site, and differences between sites for each body compartment were tested with one-way ANOVA and a multiple comparison test of means (Tukey). Prior to each analysis, all data were checked for normality using the Kolmogorov–Smirnov test and for homogeneity of variances using Cochran’s test. The effect of the site on trace element concentrations in tentacle *Symbiodinium* and anemone tissue was analyzed using the Kruskal–Wallis test. Post-hoc Mann–Whitney U-tests were run for separation of significant factors. Differences between factors were considered significant for a *p* value <0.05. Unless otherwise specified, mean values are presented ± SEM. All analyses were performed using SPSS version 20 (SPSS IBM, New York, USA).

## Results

Visual observations made during the course of sampling found anemones at both sampling sites attached to hard substratum at high abundances (of ca. 10–40 anemones m^−2^), consistent with findings by Suggett et al. (2012). At the high *p*CO_2_ site, anemones appeared to be healthy with their tentacles fully extended and without any excess amounts of mucus (Fig. 1B).

### Physicochemical properties of the seawater and suspended particles

Next to the vent the pH was significantly lowered and the *p*CO_2_ (calculated) was strongly elevated compared to typical seawater conditions that were recorded at the control site (Fig. 1A). Seawater temperature (18.5–19.5 °C) and light availability at midday (599–642 µmol quanta m^−2^ s^−1^) were similar between sites (1–2 m depth), while TA was elevated at the high *p*CO_2_ site (Fig. 1A; Table S1). The suspended particulate material collected in a net tow (> 100 µm), composed mostly of zooplankton, some of which *A. viridis* may prey upon, contained more trace elements (in weight per volume) next to the vent than at the control site (Tables 1 and S2). The degree of trace element enrichment in these particles was rather high with some trace elements enriched by 6–17 fold (Fe, Cu, As, Pb, cobalt (Co) and chromium (Cr)), while others by 2–5 fold (Mn, barium (Ba), nickel (Ni), strontium (Sr), Cd and Zn) (Table 1). Macro elements (sodium (Na), calcium (Ca), potassium (K), magnesium (Mg), boron (B) and sulfur (S)) did not show changes between sites, with the exception of phosphorus (P) (3 fold higher next to the vent).

**Table 1:** Ratios of trace element concentrations from this study and additional published data. Trace element concentration ratios in: (1) Suspended particulate material (high *p*CO_2_ site/control site), to assess the trace element content in the potential prey of *A. viridis*. Plankton hand nets (50 cm mouth diameter, 100 µm mesh size) were towed at each site for 15 min to collect plankton and other suspended particulate material from seawater; (2) Seawater, based on the report by Kadar et al. (2012). Values are ratios of trace element concentrations between: (a) Vent, vent site/control site; (b) 150 m from vent, site 150 m from vent/control site; and (c) 260 m from vent, high *p*CO_2_ site in our study/control site; (3) Sediments (high *p*CO_2_ site/control site), based on the report by Vizzini et al. (2013); (4) The seagrass *Cymodocea nodosa*. Values are ratios of trace element concentrations in: (a) epiphytes and (b) leaves.

Element	(1) Suspended particulate material	(2) Seawater			(3) Sediments	(4) *Cymodocea nodosa*	
		(a) Vent	(b) 150 m from vent	(c) 260 m from vent		(a) Epiphytes	(b) Leaves
**Trace elements**							
Fe	14	2.4	3	1.5	1.2	1.1	11
Pb	7.9	2.2	0.5	0.6	1.3	1.4	2.6
Cu	17	>11	n.d.	n.d.	1.2	1	1.7
Co	5.8	–	–	–	1.2	1.1	2
Sr	1.7	1	1	1	–	–	–
Mn	3.6	7.7	3.2	1.9	1.2	1.5	0.9
Ba	4.2	1.1	0.8	0.6	2.1	1	1
Ni	3.0	–	–	–	1.6	1.7	1
Cr	9.4	0.8	2.6	4.6	1.5	1.8	5.3
Cd	2.0	–	–	–	1.3	0.1	0.7
Zn	4.8	0.9	0.9	0.9	2.1	0.3	1.1
As	12	–	–	–	1.7	0.5	5.2
**Macro elements**							
Na	1	1	1	1	–	–	–
Ca	–	1	1	1	–	–	–
K	1	0.9	0.7	0.8	–	–	–
Mg	1	1	1	1	–	–	–
B	–	1	1	1	–	–	–
S	–	1	1	1	–	–	–
P	3.0	–	–	–	–	–	–
	This study	Kadar et al. (2012)			Vizzini et al. (2013)		

### Distribution of trace elements between the anemone pedal disc and tentacles

**Figure 2:**
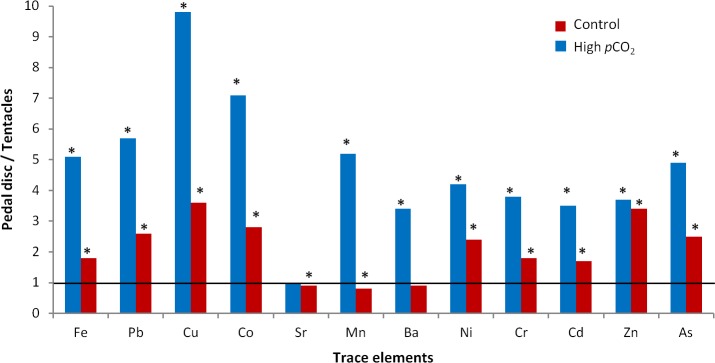
Trace element partitioning between the body compartments of *A. viridis* from sites 1 (control) and 2 (high *p*CO_2_). Mean trace element concentrations of pedal disc and tentacles are presented as ratios (pedal disc/tentacles) for each site. Asterisks indicate significant differences in mean trace element concentrations between both body compartments (Tukey, *p* < 0.05).

**Figure 3:**
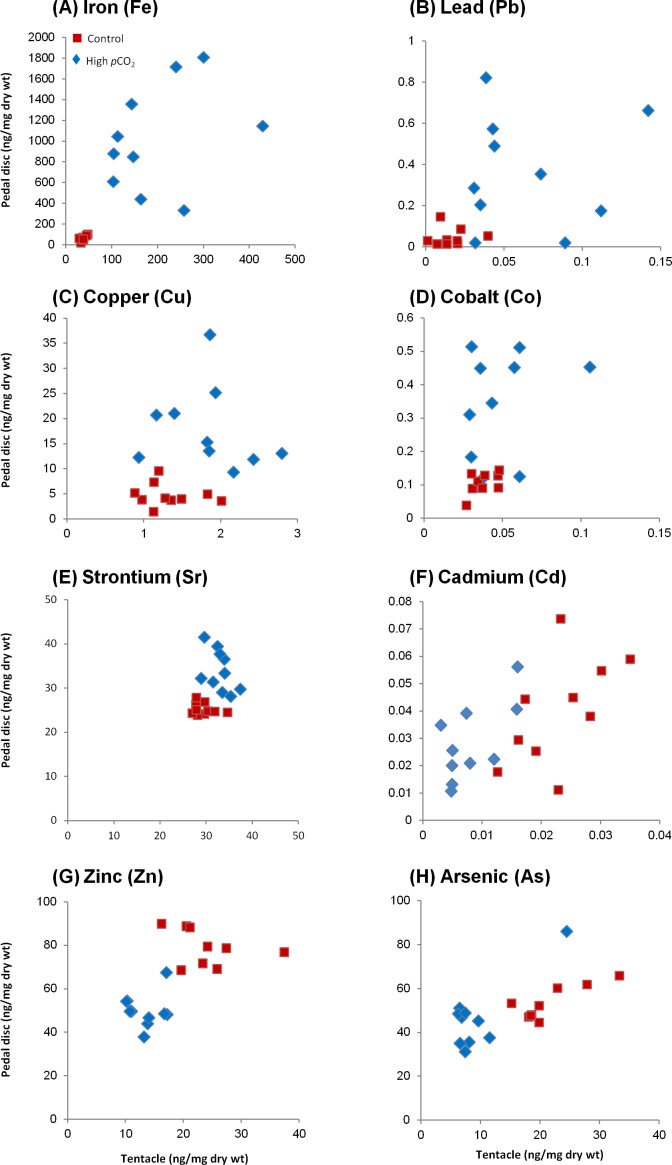
Changes in trace element concentrations of *A. viridis* between sites 1 (control) and 2 (high *p*CO_2_). Ten anemones were sampled from each site and analyzed for trace element concentrations (ng/mg dry wt) in the tentacles and pedal disc. (A) Iron; (B) lead; (C) copper; (D) cobalt; (E) strontium; (F) cadmium; (G) zinc and (H) arsenic.

Our sampling scheme provided us with a paired data set of trace element concentrations (per unit of dry weight) in the pedal disc and the tentacles of each individual anemone. Plotting the concentrations of each of the 12 tested trace elements in the pedal disc vs. that in the tentacles (10 per site), we can visualize differences among sampling sites and body compartments, as well as variability among individual anemones (Figs. 2, 3 and S1). In Fig. 3 we present concentrations of trace elements which showed the most evident changes in bioaccumulation between sites, whereas Fig. S1 shows the remainder 4 trace elements with moderate changes. Averages of these data and statistical analysis of the differences between body compartments and sites are also reported in Table 2.

**Table 2:** Trace element concentrations measured in the tentacles and pedal disc of *A. viridis* collected from sites 1 (control) and 2 (high *p*CO_2_). Mean concentrations (ng/mg dry wt), standard error (between brackets) and number of replicates (in italics) are presented for each element. Trace elements are grouped into Group I: trace elements that are accumulated in both body compartments close to the vent; Group II: trace elements that are accumulated only in the pedal disc close to the vent; and Group III: trace elements that are depleted in both body compartments close to the vent. Ratios between the high *p*CO_2_ and control sites for pedal disc and tentacle trace element concentrations (High *p*CO_2_/control) are also presented. *Letters* and *asteriks* indicate significant differences in mean trace element concentrations and ratios (Tukey, *p* < 0.05).

	Control ng/mg dry wt		High *p*CO_2_ (ng/mg dry wt)		High *p*CO_2_/control	
Element	Pedal disc	Tentacle	Pedal disc	Tentacle	Pedal disc	Tentacle
**Group I**						
Fe	67^a^	38^b^	1021^c^	195^d^	15.2^∗^	5.3^∗^
	(7)	(2)	(159)	(24)		
	*10*	*15*	*10*	*15*		
Pb	0.047^a^	0.014^b^	0.36^c^	0.048^a^	9.0^∗^	4^∗^
	(0.014)	(0.003)	(0.085)	(0.010)		
	*10*	*15*	*10*	*15*		
Cu	4.7^a^	1.2^b^	17^c^	1.6^d^	3.7^∗^	1.4^∗^
	(0.7)	(0.1)	(2.6)	(0.15)		
	*10*	*15*	*10*	*15*		
Co	0.1^a^	0.035^b^	0.34^c^	0.044^b^	3.3^∗^	1.3
	(0.0098)	(0.002)	(0.049)	(0.0055)		
	*10*	*15*	*10*	*15*		
Sr	25^a^	29^b^	33^c^	31^c^	1.3^∗^	1.1^∗^
	(0.41)	(0.77)	(1.4)	(0.73)		
	*10*	*15*	*10*	*15*		
**Group II**						
Mn	2.7^a^	3.5^b^	15^c^	2.7^ab^	5.7^∗^	0.9
	(0.19)	(0.14)	(3.2)	(0.38)		
	*10*	*15*	*10*	*15*		
Ba	1^a^	1.2^a^	3.2^b^	1^a^	3.0^∗^	0.8
	(0.2)	(0.16)	(0.68)	(0.084)		
	*10*	*15*	*10*	*15*		
Ni	0.21^a^	0.089^b^	0.35^c^	0.075^bd^	1.6^∗^	0.9
	(0.022)	(0.005)	(0.032)	(0.0078)		
	*10*	*15*	*10*	*15*		
Cr	0.39^a^	0.17^b^	0.58^c^	0.13^b^	1.5^∗^	0.7
	(0.041)	(0.029)	(0.055)	(0.018)		
	*10*	*15*	*10*	*15*		
**Group III**						
Cd	0.04^a^	0.02^b^	0.028^ab^	0.0066^c^	0.7	0.4^∗^
	(0.0061)	(0.0018)	(0.0044)	(0.0011)		
	*10*	*15*	*10*	*15*		
Zn	79^a^	22^b^	50^c^	13^d^	0.6^∗^	0.6^∗^
	(2.8)	(1.5)	(2.4)	(1)		
	*10*	*15*	*10*	*15*		
As	53^a^	20^b^	47^a^	10^c^	0.9	0.4^∗^
	(2.5)	(1.4)	(4.9)	(1.4)		
	*10*	*15*	*10*	*15*		

At the control site, most trace elements were more concentrated in the pedal disc than in the tentacles (*p* < 0.05; Table 2), with pedal disc to tentacles trace element ratios ranging between 1.6 and 3.6 (*p* < 0.05; Fig. 2). Few trace elements, including Sr, Mn and Ba were slightly enriched in the tentacles (Table 2), with pedal disc to tentacles trace element ratios of 0.8–0.9 (Fig. 2). At the high *p*CO_2_ site, all trace elements except Sr, were more concentrated in the pedal disc than in the tentacles (Table 2), and the pedal disc to tentacles trace element ratios increased to 3.4–9.8 (Fig. 2). These elevated pedal disc to tentacle ratios reflect favorable accumulation of some trace elements in the pedal disc, while for others it results from depletion of trace elements from the tentacles compared to the control site.

Based on the findings above, we compare between sites in a compartment-specific manner. Such comparisons across all tested trace elements yielded three separate behaviors (Figs. 3A–3H, S1; Table 2): (1) Group I: Trace elements that are favorably accumulated in both body compartments at the high *p*CO_2_ site (Fe, Pb, Cu, Co and Sr), (2) Group II: Trace elements that are accumulated in the pedal disc but are slightly depleted in the tentacles at the high *p*CO_2_ site (Mn, Ba, Ni and Cr), and (3) Group III: Trace elements that are depleted in both body compartments at the high *p*CO_2_ site (Zn, As and Cd). Statistical differences between sites are found for most trace elements mentioned above (*p* < 0.05), except Co and Group II elements in the tentacles, and Cd and As in the pedal disc (Table 2). The most significant change between sites was observed for Fe, which concentrations increased from 67 ± 7 to 1,021 ± 159 ng/mg dry wt in the pedal disc, and from 38 ± 2 to 195 ± 24 ng/mg dry wt in the tentacles (Table 2). Other metals in Group I, which included Pb, Cu and Co also showed substantial (3–9 fold) accumulation in both body compartments. In Group II, Mn and Ba accumulated 3–5 fold more in *A. viridis* pedal disc compared to control animals, while Ni and Cr showed very little change. However, in the tentacles, the concentrations of these trace elements were lower next to the vent than at the control site (Fig. S1; Table 2). This pattern of trace element depletion next to the vent was even more pronounced in Group III, comprising Zn, As and Cd, with lower concentrations in both body compartments at the high *p*CO_2_ site than at the control (Figs. 3F–3H; Table 2).

Macro element concentrations (Na, Ca, K, Mg, B and S) did not change in both body compartments between sites. P, however, significantly increased in the pedal disc at the high *p*CO_2_ site from 7,673 ± 179 to 8,279 ± 224 ng/mg dry wt (*p* < 0.05), whereas there was a significant decrease from 3,162 ± 168 to 2,201 ± 197 ng/mg dry wt in the tentacles (*p* < 0.05).

### Distribution of trace elements among the algae and animal tissue of the tentacles

#### Data analysis

**Figure 4:**
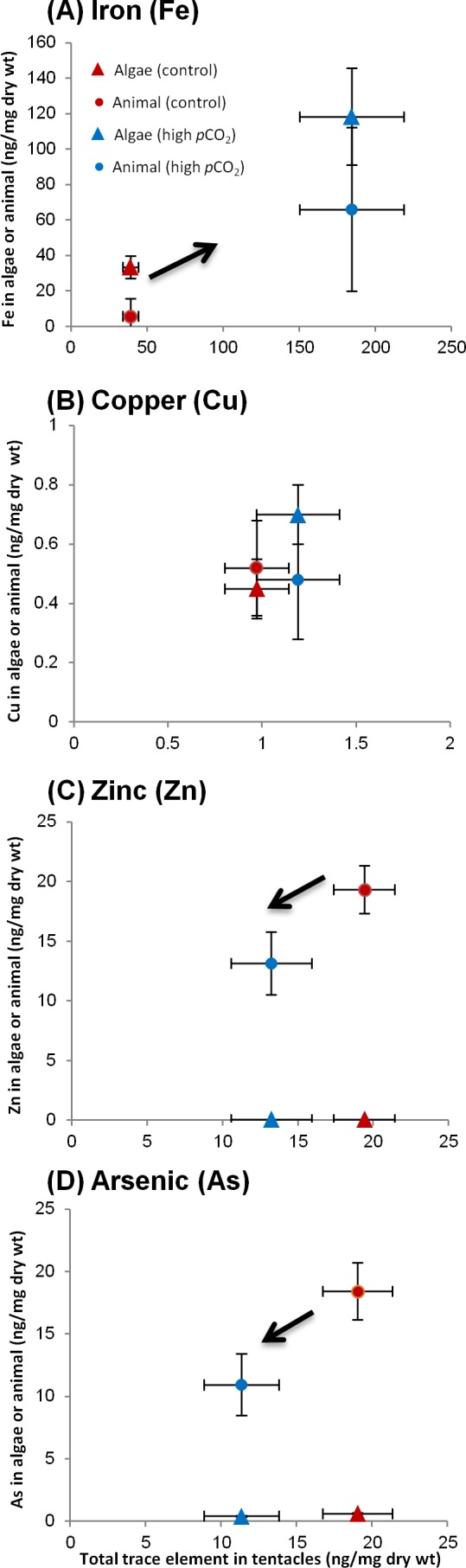
Changes in trace element partitioning in algae and animal tissue of *A. viridis* tentacles between sites 1 (control) and 2 (high *p*CO_2_). Mean trace element concentrations (ng/mg dry wt) measured in total tentacle, algae and animal host tissue fractions of: (A) Iron (high *p*CO_2_, *n* = 5; control, *n* = 4); (B) copper (both sites, *n* = 5); (C) zinc (both sites, *n* = 5) and (D) arsenic (both sites, *n* = 5). The trace element content in the animal tissue is the calculated difference between the total and the algal trace element concentrations.

Having analyzed the algae and host fractions as well as the whole tentacle, we can probe for contamination or trace element loss in the separation procedure, by comparing the sum of the separated fractions to the whole tentacle (once weight normalized). Six of the trace elements (Ni, Cr, Co, Mn, Ba and Sr) were screened out due to contamination or detection problems. In three trace elements (Fe, Pb and Cu), that were mostly associated with the algae, the sum of the separated fractions balanced out adequately with the total. In the other trace elements (Cd, Zn and As), which were mostly associated with the host, the total greatly exceeded the sum of the separated fractions, indicative of significant metal loss. Hence, we chose not to include the measured host fraction. Instead, it was calculated from the difference between the total and algal fraction (Figs. 4A–4D; Table 3). Since this is a calculated measure, we did not perform statistical analysis on these results. In addition to Table 3, we present data for several trace elements in Fig. 4 demonstrating different partitioning between algae and animal tissue.

**Table 3:** Trace element partitioning between algae and animal tissue in the tentacles of *A. viridis* collected from sites 1 (control) and 2 (high *p*CO2). For each site we analyzed the trace element concentrations in total tentacle, algae and animal host tissue. Mean concentrations (ng/mg dry wt), standard error (between brackets) and number of replicates (in italics) are presented for each element. Host tissue values are calculated as the difference between total and algal trace element concentrations. The partitioning of trace elements between the algae (Alg) and the animal (Ani) fractions are presented as percentage of the tentacles’ total trace element concentration. *Letters* indicate significant differences in mean trace element concentrations (Mann–Whitney, *p* < 0.05).

							Fraction from total			
	Control ng/mg dry wt			High *p*CO_2_ (ng/mg dry wt)			Control (%)		High *p*CO_2_ (%)	
Element	Total	Algal	Animal	Total	Algal	Animal	Alg	Ani	Alg	Ani
Fe	39^a^	33^a^	5.7	184^b^	118^b^	66	85	15	64	36
	(5.1)	(6.3)	(10)	(34)	(27)	(46)				
	*4*	*4*	*4*	*5*	*5*	*5*				
Pb	0.0082^a^	0.005^a^	0.0031	0.022^b^	0.0068^a^	0.015	63	37	33	67
	(0.001)	(0.0022)	(0.0021)	(0.013)	(0.001)	(0.013)				
	*4*	*4*	*4*	*4*	*4*	*4*				
Cu	0.97^a^	0.45^b^	0.52	1.19^a^	0.7^ab^	0.48	46	54	60	40
	(0.17)	(0.1)	(0.16)	(0.22)	(0.1)	(0.2)				
	*5*	*5*	*5*	*5*	*5*	*5*				
Zn	19^a^	0.086^b^	19	13^c^	0.075^b^	13	0	100	1	99
	(2)	(0.015)	(2)	(2.6)	(0.018)	(2.6)				
	*5*	*5*	*5*	*5*	*5*	*5*				
As	19^a^	0.62^b^	18	11^c^	0.41^d^	10	3	97	4	96
	(2.3)	(0.052)	(2.2)	(2.4)	(0.051)	(2.4)				
	*5*	*5*	*5*	*5*	*5*	*5*				
Cd	0.013^a^	0.0012^b^	0.012	0.0037^c^	0.0016^b^	0.002	9	91	45	55
	(0.001)	(0.00037)	(0.0012)	(0.0011)	(0.0004)	(0.0014)				
	*5*	*5*	*5*	*5*	*5*	*5*				

#### Trace element distributions among fractions

At the control site the trace element which associated most strongly with the algae was Fe, with 85% of the Fe in the algal fraction (Table 3). Pb and Cu were distributed more or less evenly between the algae and the tissue at the control site, while Zn, As and Cd were found predominantly (90–100%) in the host tissue (Table 3). Next to the vent, the tentacles had accumulated Fe, Cu, and Pb, but lost Zn, Cd and As compared to the control site (*p* < 0.05 for whole tentacle trace element concentrations; Tables 2 and 3). Iron, the trace element that accumulated to the greatest extent near the vent, increased significantly in the algae from 33 ± 6.3 to 118 ± 27 ng/mg dry wt (Fig. 4A). However, the animal tissue accumulated more Fe compared to the algal fraction, and subsequently only 64% of the tentacle Fe appears in the algal fraction at the vent site (Fig. 4A; Table 3). Similarly, Pb increased substantially next the vent, but accumulated mostly in the animal tissue and not in the algae, subsequently accounting for ∼70% of the tentacle Pb (compared to ∼40% at the control site; Table 3). Copper, on the other hand, was preferably accumulated in the algae, although not significantly, accounting at the vent site for 60% of the tentacle Cu (compared to 46% at the control site; Fig. 4B). Zn and As were both depleted next to the vent, but remained predominately in the animal tissue (Figs. 4C–4D; Table 3). There was a significant decrease of As in the algae from 0.62 ± 0.052 to 0.41 ± 0.051 ng/mg dry wt (*p* < 0.05). Cd, however, was lost predominantly from the host tissue and the low remaining Cd was evenly distributed between the algae (45%) and host tissue (55%) (Table 3).

## Discussion

The shallow CO_2_ vents at Levante Bay of Vulcano Island are intensively studied within the frame of OA research. Given the intrinsic geochemical complexity of the location, we studied the combined effects of trace element exposure and elevated *p*CO_2_ on the anemone *A. viridis*. The present study provides important insights into the performance of *A. viridis* under natural (control) and enriched trace element conditions. Collectively, our data shows that trace elements are differentially accumulated in *A. viridis* between ambient (control) and low pH/high *p*CO_2_ environments, which confirms our hypothesis (Figs. 2–4; Tables 2 and 3). In the ensuing discussion we first address the “trace element environment” the anemones experience nearby the vent and then explore the accumulation or depletion patterns of selected trace elements in different body compartments and between sites.

### The “trace element environment” nearby the vent

In order to analyze the trace element profiles in *A. viridis* growing at normal and high *p*CO_2_ sites, it is first necessary to compare their “trace element environments”. Anemones can accumulate their trace elements from seawater, sediments, and suspended particulate material (including prey). The trace element accumulation pathways consist of: absorbance across the body surface, ingestion of particles, and/or active predation (Harland & Nganro, 1990; Harland, Bryan & Brown, 1990).

Previous reports have indicated that the seawater close to the CO_2_ vent was enriched in Fe, Cu, Zn, Cr, and Mn (Table 1). Most of these metals were present as organic and/or inorganic complexes in the nano-particles size range (<100 nm fraction), while Fe was mostly associated with larger (>100 nm) colloids or particles (Kadar et al., 2012; Boatta et al., 2013). Fe, Mn and Cr show the most pronounced increase at the high *p*CO_2_ site, while Sr, Ba and Zn remain unchanged (Table 1). Seawater concentrations of other trace elements included in our study, namely Cd and As, were not analyzed. Sediments collected from the high *p*CO_2_ site were enriched in trace elements compared to the control site (Vizzini et al., 2013). Elements showing the highest enrichments were Ba, Ni, Zn and As (Table 1). Next to the vent, the low pH increases the solubility of some metals in the sediments, resulting in increased bioavailability and/or toxicity (Roberts et al., 2013). Similarly, the low pH and other elements released by the vents are likely to alter the speciation of trace elements in the seawater and subsequently their availability to the anemones (Byrne, Kump & Cantrell, 1988; Millero et al., 2009). While the study by Kadar et al. (2012) provided some information on the speciation of trace elements next to the vent, we currently cannot link the speciation of trace elements to their availability to anemones, which bioaccumulate trace elements through multiple pathways.

Here we added the analysis of trace elements in particles larger than 100 µm, which presumably represent zooplankton and other particles that the anemone can ingest or prey upon. All tested trace elements were substantially enriched in the particles collected next to the CO_2_ vent compared to the control site (by a factor of 2–18; Table 1). All in all, the above mentioned data, both from the literature and this study, indicates that the “trace element environment” next the CO_2_ vent, that is seawater, sediments and suspended particulate material, is different to the control site. Subsequently, regardless of the trace element uptake pathways in the anemones, their trace element content likely differs between sites.

### Trace element accumulation patterns at the high *p*CO_2_ site (highlighting Fe, Cu and Pb)

Of the 12 trace elements tested, the concentrations of five elements (Fe, Cu, Pb, Co and Sr) increased in both body compartments at the high *p*CO_2_ site compared to the control site (Group I; Table 2), while four other trace elements (Mn, Ni, Ba and Cr) were elevated only in the pedal disc at the high *p*CO_2_ site (Group II; Table 2). An increase in trace element concentrations of anemones (whole body) exposed to elevated loads of dissolved trace elements in the water was frequently observed in laboratory studies (Harland & Nganro, 1990; Harland, Bryan & Brown, 1990; Mitchelmore et al., 2003; Mitchelmore, Ringwood & Weis, 2003; Main, Ross & Bielmyer, 2010; Brock & Bielmyer, 2013). Additionally, differences in the overall metabolic activity of the anemones under elevated *p*CO_2_ (Towanda & Thuesen, 2012; Suggett et al., 2012) may contribute to increased trace element bioaccumulation. This may be due to the higher rates of respiration in the more metabolically active anemone (Suggett et al., 2012), which directly affects feeding rate and thus the uptake of waterborne trace elements (Zamer, 1986). It is commonly accepted that the excess trace elements are kept in a “chemically safe” form through binding by proteins or other organic molecules and/or formation of metal granules, and in some cases are stored in a specialized organ (i.e., hepatopancreas or digestive gland in crustaceans, Depledge & Rainbow, 1990; Rainbow, 1990; Rainbow, 1993). Our measured trace element profiles in different body compartments and sites may serve to illustrate where trace elements are stored. Below we focus on three trace elements—Fe, Pb, and Cu, which were found in high concentrations in suspended particulate material, as well as seawater and sediments nearby the vent (Tables 1 and S2).

#### Iron

As suggested by Millero et al. (2009), a pH decrease from 8.1 to 7.4 (as in the high *p*CO_2_ site in our study) is responsible for enhanced Fe solubility of 40%, causing an increase in Fe bioavailability in the water column. Accordingly, a high seawater concentration of dissolved Fe was reported close to the primary CO_2_ vent (Kadar et al., 2012; Boatta et al., 2013), as well as in suspended particulate material collected in this study (14 fold higher compared to the control site; Table 1). Indeed, the most significant change at the high *p*CO_2_ site was found for Fe, which accumulated in both the anemone tentacles and pedal disc (5 and 15 fold, respectively; Table 2). The pedal disc preferably accumulated Fe at both sites compared to the tentacles, but to a greater degree at the high *p*CO_2_ site (Figs. 2 and 3A). The concentration of Fe in the different body compartments and sites ranges from ∼40 to 1,000 ng/mg dry wt and is the highest of the trace elements we studied. In the tentacles, Fe was found predominantly in the algae (85% of total) at the control site. Next to the vent, the partitioning changed and the algae accounted for only ∼65% of the total tentacle Fe (Fig. 4A; Table 3). The high Fe values in the pedal disc point to its possible role in iron storage. While Fe is often limiting phytoplankton growth in the ocean (Sunda & Huntsman, 1997), it is unlikely that these coastal anemones ever experience Fe limitation (Lis & Shaked, 2009). Hence the Fe reserves in the pedal disc are probably not required to maintain the anemone Fe demands. Rather, the suggested Fe compartmentalization in the pedal disc can be viewed as a means to keep the Fe localized and in an inactive form, where it can not react with oxygen to generate harmful reactive oxidative species (ROS) (Lesser, 2006). Iron is the most important trace element for algal growth, specifically utilized at high quantities by the photosynthetic apparatus (Falkowski, Barber & Smetacek, 1998; Davey & Geider, 2001). At the control site, the preferable partitioning of the iron to the algal fraction of the tentacles seems to reflect the high Fe requirements of the symbiotic algae. Next to the vent, the Fe concentrations in the algal fraction increased, presumably enabling them to maintain elevated photosynthetic rates as reported at this site (Suggett et al., 2012). The Fe requirements of the algae at the same site were probably met, as Fe accumulates in the host tissue to a larger extent compared to the control site (Table 3). In another study in Levante Bay, the concentration of Fe was 10-fold higher in the leaves of the seagrass *Cymodocea nodosa* at the high *p*CO_2_ site compared to the control (Vizzini et al., 2013) (see Table 1). This adds additional perspective on other organisms exposed to the same conditions and emphasizes that trace element bioaccumulation at this site occurs across a broad biological scale.

#### Lead

In addition to the elevated seawater concentration reported for Pb nearby the vent (Kadar et al., 2012) and in suspended particulate material (Table 1), this potentially toxic trace element in seawater increases by 100% in free ion concentration when pH decreases from 8.1 to 7.4, subsequently rendering it more bioavailable (Millero et al., 2009). Accordingly, analysis of Pb concentrations in the leaves and epiphytes of *C. nodosa* nearby the vent revealed an increase compared to control samples (Vizzini et al., 2013) (see Table 1). Similarly, our results show significant accumulation of Pb next to the vent in both *A. viridis* body compartments, with a higher concentration in the pedal disc compared to the tentacles (Table 2). In the tentacles, Pb is more prevalent in the algal fraction at the control site (63% of total; Table 3). Next to the vent, the excess Pb that accumulated in the tentacles is partitioned predominately into the host tissue (67% of total; Table 3). Unlike Fe and Cu, Pb has no known requirement in the animal and is considered highly detrimental to the organism’s health (Rainbow, 2002). Lacking previous studies of Pb in anemones, we cannot deduce whether the measured concentrations may impose toxicity. It is, however, interesting to note that Pb is found with the lowest concentrations (∼0.1–0.4 ng/mg dw) of the three trace elements discussed here, possibly reflecting its non-essentiality and potential toxicity. As with the other trace elements, the preferential accumulation of Pb in the pedal disc indicates that this body compartment serves as a storage compartment for trace elements (Fig. 2; Table 2).

#### Copper

Cu concentration in suspended particulate material was 17 fold higher near the vent compared to control conditions, the highest ratio measured out of the trace elements we examined (Table 1). This is in accordance with substantial enrichment in the seawater, as measured by Kadar et al. (2012) (Table 1). As seawater pH decreases there is also an increase in free copper ion concentration and hence copper bioavailability (Millero, Santana-Casiano & González-Dávila, 2010). Richards et al. (2011) predicted an increase of 115% in free copper ions over the next 100 years in coastal waters as a result of declining seawater pH (Pascal et al., 2010; Fitzer et al., 2013; Lewis, Clemow & Holt, 2013). In the area close to the vent in Levante Bay, Vizzini et al. (2013) found Cu was elevated in the leaves of *C. nodosa* (Table 1). Our results also support the changes in Cu ion speciation and rising bioavailibility. Similar to Fe and Pb, Cu accumulated next to the vent in both body compartments and was found predominantly in the pedal disc rather than the tentacles (Figs. 2 and 3C; Table 2). In the tentacles it was evenly distributed between the algal and animal fractions at the control site, while next to the vent the algal fraction gained a slight dominance over the host fraction (Fig. 4B; Table 3). Cu has a key metabolic role in marine invertebrates’ physiology (White & Rainbow, 1985), but at high levels it can be very toxic (Rainbow, 1997). Cu was found in tentacle nematocysts, dense granules in the cytoplasm of the nematocytes and other ectodermal cells possibly involved in cell maturation (Gupta & Hall, 1984). The algal symbionts were found to contain significant amounts of Cu at ambient conditions and under increased Cu exposure (Harland & Nganro, 1990), in agreement with our data. Laboratory studies that exposed anemones to elevated but sub-lethal Cu concentrations reported increased body loads of 20–75 ng/mg dry wt (Harland & Nganro, 1990; Mitchelmore et al., 2003; Main, Ross & Bielmyer, 2010; Brock & Bielmyer, 2013). These body loads are higher than those measured in our study of 1–18 ng/mg dry wt (Table 2), indicating that the anemones are not experiencing Cu toxicity. Indeed, next to the vent the anemones were abundant and functional (Borell et al., 2014), and even showed improved photosynthetic capabilities, probably due to the high *p*CO_2_ levels (Suggett et al., 2012).

### Trace element depletion patterns at the high *p*CO_2_ site (highlighting Zn, Cd and As)

In contrast to the trace element enrichment in the suspended particulate material and sediments next to the vent (Table 1), the concentrations of some of the trace elements in the anemones were lower at the high *p*CO_2_ site than in those of the control site. Three trace elements - Zn, Cd and As, showed the most pronounced depletion in both the tentacles and the pedal disc (Group III; Table 2). Four other trace elements - Mn, Ni, Ba and Cr, were depleted to a lesser extent and in the tentacles only (Group II; Table 2). Intrigued by this unexpected depletion, we tested how the trace elements in the tentacles partition between the algae and the host tissue. Since anemones were shown to expel their algae when subjected to trace element stress (Harland & Nganro, 1990; Mitchelmore et al., 2003), trace elements that occur predominately in the algae may be removed via this mechanism. The trace element depletion in the anemones may reflect their efficient elimination system, as was previously observed in laboratory studies during the recovery stages of anemones from high trace element loads (Mitchelmore et al., 2003; Brock & Bielmyer, 2013). It is also possible that changes in ion speciation due to the low pH diminish the bioavailability of these trace elements (Millero et al., 2009). Below, we explore the profiles of Group III elements among body compartments and sites and briefly speculate on their modes of regulation.

#### Zinc and Cadmium

Although the concentration of Zn and Cd was enhanced in the low pH environment in both suspended particulate material and sediments (Table 1), their chemical form may render them less bioavailable as their free-ion concentration may decrease or be unaffected (Millero et al., 2009; Lacoue-Labarthe et al., 2009; Lacoue-Labarthe et al., 2011; Lacoue-Labarthe et al., 2012; Pascal et al., 2010). As with most other trace elements in this study, Zn and Cd were favorably accumulated in the pedal disc compared to the tentacles at the control site (Table 2). Next to the vent, their concentrations dropped in both body compartments, but to a greater degree in the tentacles. Interestingly, in the study by Vizzini et al. (2013), Cd was also depleted in both the leaves and epiphytes of *C. nodosa* sampled from the high *p*CO_2_ site compared to the control site. Zn, on the other hand, only decreased in the epiphytes of the seagrass at the high *p*CO_2_ site (Table 1). Both trace elements were predominantly found in the animal fraction of the tentacles under ambient conditions (100% for Zn and 91% for Cd; Table 3). Next to the vent, where their tissue concentrations were significantly lower, Zn maintained the same partitioning but Cd changed (with only 55% in the host tissue). Although discussed jointly, these trace elements vary in their use. Cd is considered non-essential, while Zn plays multiple biochemical roles as a co-factor in enzymes (e.g., in carbonic anhydrase), in DNA (Zn fingers) and more (Simkiss, 1979; Viarengo & Nott, 1993; Scott et al., 2013). At high concentrations these trace elements may become toxic (Rainbow, 1997). Are the anemone concentrations of Zn and Cd from the control site high enough to be toxic? Comparison with laboratory studies shows that this is not the case, with Zn and Cd anemone body loads of 20–80 and 0.02–0.04 ng/mg dry wt, respectively (Table 2). These values are even lower than typical anemone Zn and Cd concentrations of 100–200 and ∼2 ng/mg dry wt, respectively, observed in control experiments without added metals (Brock & Bielmyer, 2013). Zn and Cd share some chemical resemblance and are often transported to and from the cells via the same transport pathways. For instance, Cd uptake in *A. viridis* was reduced in the presence of Zn, when the Zn:Cd ratio was greater than 10:1 (Harland, Bryan & Brown, 1990). In the mussel *Perna viridis* a decrease in Zn influx with increasing Zn exposure was shown to cause a reduction in Cd influx (Blackmore & Wang, 2002; Shi & Wang, 2004). A slowdown in uptake of both trace elements may be put forward as a partial explanation of our findings. Another possibility of trace element inter-replacement in different proteins may also explain the drop in Zn and Cd content. Cu, a trace element that accumulated next to the vent is known to replace Zn in various proteins, and may play some role here as well (Kägi & Kojima, 1987; Yang & Thompson, 1996). It is also possible the unique water chemistry near the vent, including lower pH and H_2_S, may influence the influx and outflux of trace elements due to changes in cell membrane permeability in the anemones. The observed patterns of reduced trace element concentrations may reflect impaired uptake and lower requirement, but may also be indicative of active trace element excretion. Anemones were reported to lose some of their trace elements by releasing their symbiotic algae from the tentacles (Harland & Nganro, 1990). Since Zn and Cd are associated predominantly with the host tissue (Table 3), this pathway (if operates) is not an effective means of depleting their concentrations. Nonetheless, it is likely that the anemones operate additional pathways for trace element removal.

#### Arsenic

Volcanism and related hydrothermal systems are important sources of As to the environment (Aiuppa et al., 2006). High As concentrations (up to about 7000 mg/L) were reported in the volcanic groundwaters of Vulcano Island (Aiuppa et al., 2003). Indeed, we detected a considerable increase of As in suspended particulate material (>100 µm) close to the vent (12 fold higher; Table 1). However, in contrast to the external elevated As, the anemones at this site contained lower As concentrations compared to the control anemones (Figs. 2G and 3; Table 2). Correspondingly, Vizzini et al. (2013) found that the concentration of As was decreased in epiphytes of *C. nodosa* taken from the high *p*CO_2_ site compared to control samples (by 50%), although there was enhanced accumulation in the leaves (see Table 1). Since As is structurally analogous to phosphorus, it can be taken up by phosphate transport systems (Nies & Silver, 1995). These elements may thus interfere with each other by competing for the same transport pathway in *A. viridis* (P is also depleted next to the vent in the tentacles; see “Distribution of trace elements between the anemone pedal disc and tentacles”). Similar to Cd and Zn, As is associated with the host tissue (Fig. 4D; Table 3), and hence its removal mechanism cannot involve symbiont release. The pathways and ecological benefits of depleting As (as well Zn and Cd) concentrations from the anemone tentacles and pedal disc are mostly unknown at current. However, the data for Group III elements, together with that of the other depleted trace elements (in Group II), suggest that the anemones tightly regulate their body loads. As is also more concentrated in the pedal disc than in the tentacles at both sites (Fig. 2), further supporting the notion of this body compartment as a storage/detoxification site.

### Environmental implications

The strong gradients in trace element concentrations under OA-like conditions in the relatively small spatial scale of Levante Bay enabled us to detect distinct patterns of trace element accumulation and depletion in *A. viridis* from contrasting environments. These, in turn, may help predict the ability of anemones to adapt to such conditions, as coastal marine invertebrates will have to cope with OA together with other anthropogenic stressors such as chronic environmental pollution in the future (Lewis, Clemow & Holt, 2013). It was not within the scope of our study to discern whether trace element uptake is biologically-driven or based on physicochemical changes of the metals and metalloids in the water column. Our results, however, demonstrate the potential resilience of *A. viridis* to acidification combined with trace element pollution, and moreover, they seem to thrive in this environment (Suggett et al., 2012). Despite potentially harmful levels of trace elements within the region of our study site (260 m from the vent), we found no apparent toxicity to the anemones although there was clear bioaccumulation. The conditions of high *p*CO_2_/low pH and elevated trace elements may pose some metabolic costs to the anemones. Animals subjected to high *p*CO_2_/low pH are required to compensate for acid–base imbalance in intra- and extracellular spaces (Fabry et al., 2008). Anemones subjected to high trace element fluxes are forced to invest in metal regulation and compartmentalization (Harland & Nganro, 1990; Mitchelmore et al., 2003; Brock & Bielmyer, 2013). On the other hand, high *p*CO_2_ conditions are metabolically favorable for anemones due to increased photosynthesis (Towanda & Thuesen, 2012). Although we are unable to determine these metabolic costs, we assume gain outweighs costs in this environment based on their prevalence and large dimensions (Suggett et al., 2012). We think these are valuable considerations that merit further study for predicting anemones’ performance under increasing trace element loads and low pH. To our knowledge, this is the first study performed *in situ* in an effort to better understand and describe the potential impact of trace element pollutants under OA conditions on an ecologically important coastal invertebrate. This study is unique in terms of long-term chronic exposure to multiple trace elements and high *p*CO_2_/low pH conditions, and as such bears significant implications to current predictions of OA impacts on coastal marine communities.

## Supplemental Information

10.7717/peerj.538/supp-1 Table S1 Physicochemical properties of the seawater Measurements of pH, TA, temperature, calculated *p*CO_2_ and midday (12:00–13:00 h) light intensities at stations 1 (control) and 2 (high *p*CO_2_). Both sites were at 1–2 m depth. Data represent the mean value (± SD).

10.7717/peerj.538/supp-2 Table S2 Trace element content of suspended particulate material collected in the water column at sites 1 (control) and 2 (high *p*CO_2_) Suspended particulate material was sampled to assess the trace element content in the potential prey of *A. viridis*. Plankton hand nets (50 cm mouth diameter, 100 µm mesh size) were towed at each site for 15 min tocollect plankton and other suspended particulate material from seawater. The measured concentrations (ppb) are presented per tow.

10.7717/peerj.538/supp-3 Figure S1 Changes in trace element concentrations of *A. viridis* between sites 1 (control) and 2 (high *p*CO_2_) Ten anemones were sampled from each site and analyzed for trace element concentrations (ng/mg dry wt) in the tentacles and pedal disc. (A) Manganese, (B) barium, (C) nickel, and (D) chromium.
