# Supplementary material for: Trace element profiles of the sea anemone Anemonia viridis living nearby a natural CO2 vent
Source: PeerJ. 2014 Sep 9;2:e538. doi: 10.7717/peerj.538 (PMC4168758; doi:10.7717/peerj.538)
Supplement: Table S1 — Measurements of pH, TA, temperature, calculated pCO2 and midday (12:00–13:00 h) light intensities at stations 1 (control) and 2 (high pCO2). Both sites were at 1–2 m depth. Data represent the mean value (± SD). [file peerj-02-538-s001.docx]

| Station | pH (NBS) | TA (μEq L^-1^) | *p*CO2  (μatm) | Temperature  (°C) | Light  (μmol quanta m^-2^ s^-1^) |
| --- | --- | --- | --- | --- | --- |
| High *p*CO_2_ | 7.44  (0.26) | 2554  (47) | 3269  (836) | 19.12  (0.69) | 599  (39) |
| Control | 8.12 (0.02) | 2471 (10) | 463  (33) | 19.14 (0.63) | 642  (91) |
