# Supplementary material for: Trace element profiles of the sea anemone Anemonia viridis living nearby a natural CO2 vent
Source: PeerJ. 2014 Sep 9;2:e538. doi: 10.7717/peerj.538 (PMC4168758; doi:10.7717/peerj.538)
Supplement: Table S2 — Suspended particulate material was sampled to assess the trace element content in the potential prey of A. viridis. Plankton hand nets (50 cm mouth diameter, 100 µm mesh size) were towed at each site for 15 min tocollect plankton and other suspended particulate material from seawater. The measured concentrations (ppb) are presented per tow. [file peerj-02-538-s002.docx]

| Element | Control  ppb/tow | High *p*CO_2_  ppb/tow |  |
| --- | --- | --- | --- |
| Fe | 28950 | 403840 | **Group I** |
| Pb | 13 | 107 |  |
| Cu | 133 | 2239 |  |
| Co | 8.9 | 51 |  |
| Sr | 5180 | 9053 |  |
| Mn | 1629 | 5913 | **Group II** |
| Ba | 141 | 590 |  |
| Ni | 11 | 32 |  |
| Cr | 13 | 123 |  |
| Cd | 0.59 | 1.2 | **Group III** |
| Zn | 85 | 407 |  |
| As | 134 | 1608 |  |
