# Supplementary figures and images for: Trace element profiles of the sea anemone Anemonia viridis living nearby a natural CO2 vent
Source: PeerJ. 2014 Sep 9;2:e538. doi: 10.7717/peerj.538 (PMC4168758; doi:10.7717/peerj.538)

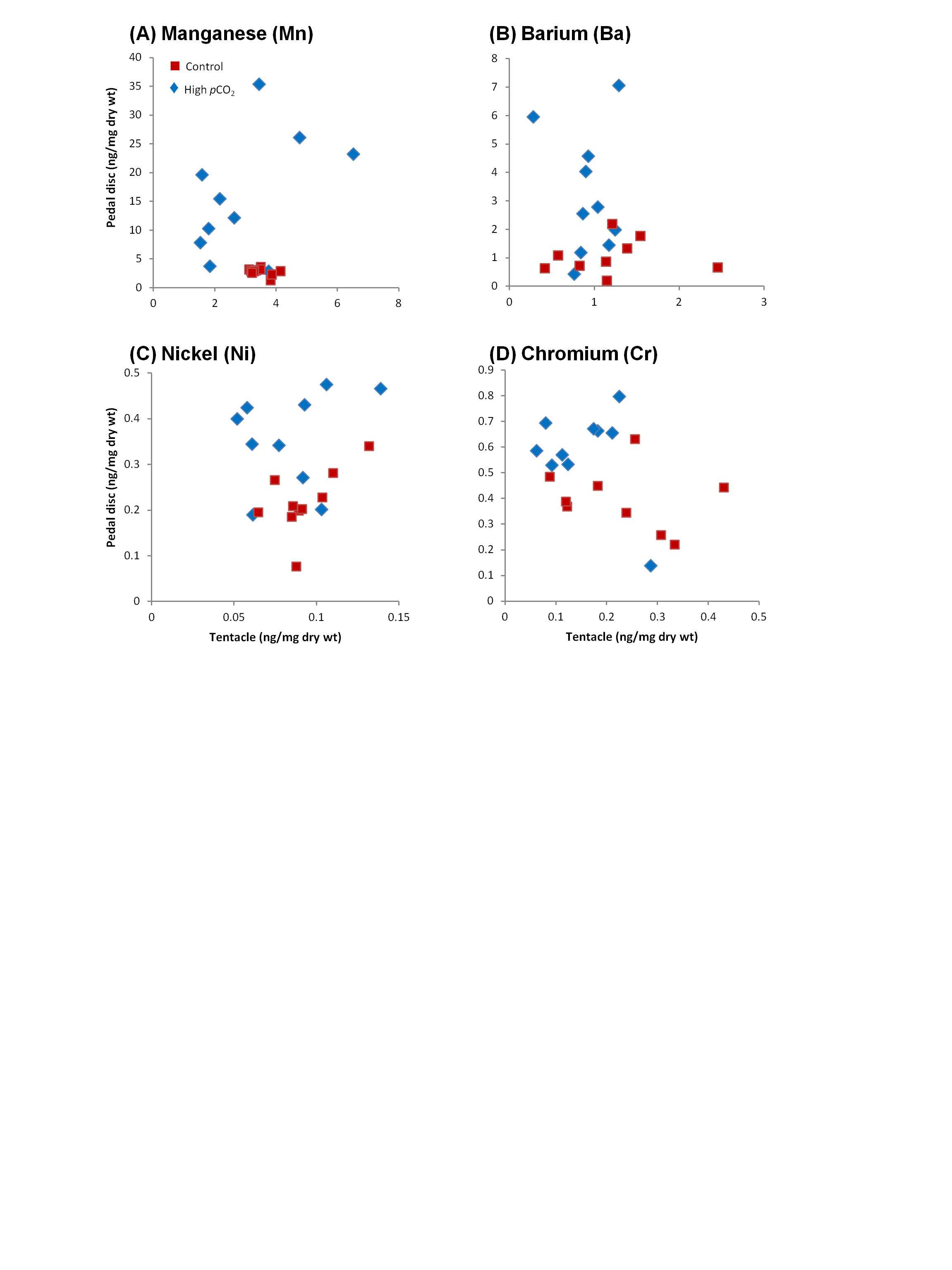

Supplement: Figure S1 — Ten anemones were sampled from each site and analyzed for trace element concentrations (ng/mg dry wt) in the tentacles and pedal disc. (A) Manganese, (B) barium, (C) nickel, and (D) chromium. [file peerj-02-538-s003.png]
